# Chest pain and ST-segment elevation in a patient with polymyositis: a case report

**DOI:** 10.1186/1757-1626-2-84

**Published:** 2009-01-23

**Authors:** Pavlos N Stougiannos, Dimitrios Z Mytas, Andreas A Katsaros, Apostolos T Kakkavas, Aristides E Androulakis, Ioannis E Kallikazaros, Dimitrios N Chrissos

**Affiliations:** 1 Department of Cardiology, Hippokration Hospital, Athens, Greece, 114, Vas. Sofias Street, 11527 Athens, Greece; 2 Department of Cardiology, Panarkadikon Hospital, Tripoli, Greece, End of Red Cross Street, 22100 Tripolis, Greece

## Abstract

**Background:**

Cardiac involvement in patients with polymyositis is well-documented and includes myocarditis, coronary arteritis, pericarditis, valvular dysfunction and arrhythmias.

**Case report:**

There are only few reports of acute myocarditis in patients with polymyositis and, although it usually follows a chronic, mild course, it may occasionally become life-threatening. We describe the case of a 36-year-old young woman suffering from polymyositis who presented with clinical signs and symptoms mimicking an ST Elevation Acute Coronary Syndrome. The atypical features of the pain, the young age of the woman, the lack of significant cardiovascular risk factors and the medical history of an autoimmune disease, led us to reconsider our initial diagnosis towards the presence of focal myocarditis.

**Conclusion:**

We describe our diagnostic approach and comment on our speculations and decisions about the treatment of such a life threatening event.

## Background

Myocarditis is a clinical entity with a great variety of clinical manifestations, which may range from the totally asymptomatic state, in the less severe cases, to the severely ill, haemodynamically-compromised patient, in the case of fulminant myocarditis.[1-3] Sometimes it can demonstrate clinical, electrocardiographic and echocardiographic abnormalities simulating an acute myocardial infarction, from which it may be difficult to be differentiated. [4-6] We present the case of a young woman who was admitted to our department with symptoms and signs mimicking an acute myocardial infarction, but who finally proved to suffer from myocarditis.

## Case presentation

A 36-year-old caucasian woman was admitted to our hospital due to a moderate to severe retrosternal pain, commencing 24 hours ago, radiating to the back and being aggravated with deep breathing. There was no fever, history of recent viral infection or any other accompanying symptoms. The woman had been suffering for the last 5 months from a low-grade fever, diffuse myalgias and worsening fatigue. She had been fully investigated during the previous month and was considered to suffer from an autoimmune disease, yet no definite proof was available. While waiting for the results of a skin-muscle-vessel and liver biopsy she was empirically set on corticosteroids and was discharged from the hospital. A few days later she presented to us with the previously described pain. From her medical history she had no significant cardiovascular risk factor.

**Figure 1:**
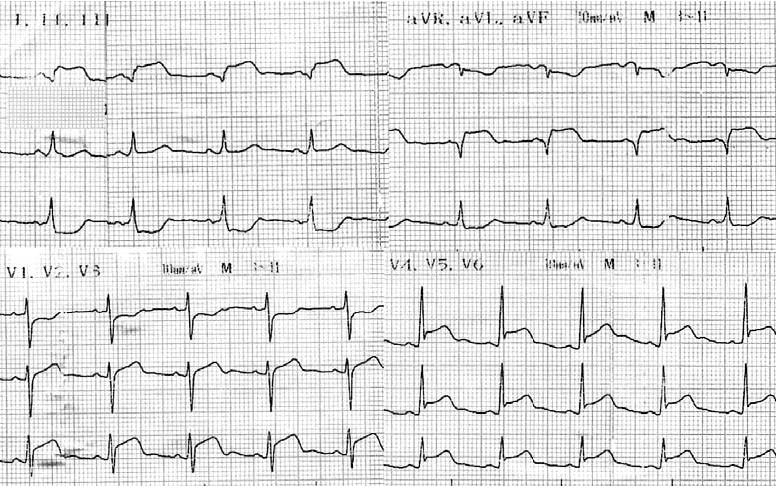
**Electrocardiogram on admission**.

On admission she looked ill. She had no dyspnoea and from her physical examination she had sinus tachycardia, normal first and second heart sounds, a fourth heart sound, a mild apical mid-systolic murmur, no pericardial friction rub and clear lung fields. On chest x-ray the heart size was normal and there were not any signs of pulmonary congestion. The admission ECG revealed sinus rhythm, normal QRS axis, a Q-wave at I, aVL and ST-segment elevation at leads I, aVL, V_3_-V_6 _with reciprocal ST-segment depression at leads III, aVF (figure 1). Cardiac enzyme levels (troponin, creatin phosphokinase-MB) were increased. Echocardiography showed a left ventricle with normal internal dimensions, an apparent regional hypokinesia of the posterior, the middle and apical segments of the lateral wall and a mildly compromised global systolic function.

The electrocardiographic and echocardiographic findings, as well as the enzymic activity, were typical of an acute myocardial infarction. However, the young age of the patient, the atypical features of chest pain, the lack of significant risk factors for coronary artery disease and the underlying autoimmune disease, led us to reconsider our diagnosis towards that of acute focal myocarditis. After a lot of consideration, we decided to initiate high-dose intravenous corticosteroid treatment, instead of immediate reperfusion with intravenous thrombolysis (as emergent coronary angiography and possible primary angioplasty were not available in our setting).

**Figure 2:**
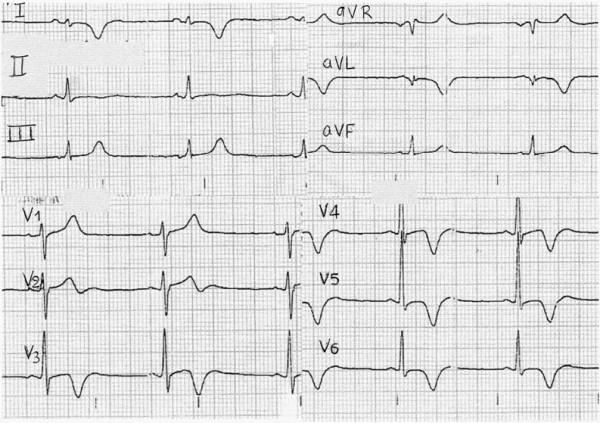
**Electrocardiogram ten days later**.

**Figure 3:**
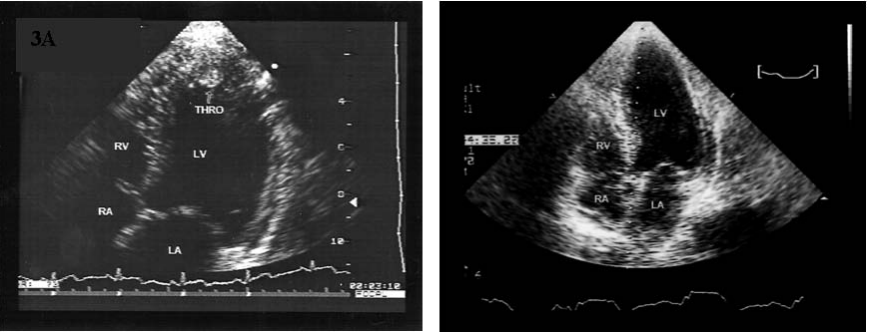
**A. Apical four chamber view**. It depicts a thrombus in the apex (arrows) Abbreviations: LV:left ventricle, RV: right ventricle, LA: left atrium, RA: right atrium, THRO: thrombus. B. The thrombus has disappeared after anticoagulant therapy. Abbreviations: as in figure 3A.

On the following days ST-segment elevations gradually decreased and negative T-waves appeared at the same leads, following the pattern of acute myocardial infarction (figure 2). However, on the third day of hospitalization, a second echocardiogram revealed the presence of a mural thrombus attached to the previously recognized hypokinetic area (figure 3A). Antithrombotic therapy was initiated (with low molecular weight heparin and acenocoumarol) and three days later the thrombus gradually disappeared (figure 3B) without any embolic sequela.

Serial echocardiographic assessment showed that the segmental wall motion abnormality gradually improved and ten days later the global contractility of the left ventricle were normal. Similarly, the cardiac enzymes that were elevated on admission returned to normal, following the pattern of an acute myocardial infarction. The patient underwent coronary angiography, which revealed normal coronary arteries and normal systolic function of the left ventricle.

**Figure 4:**
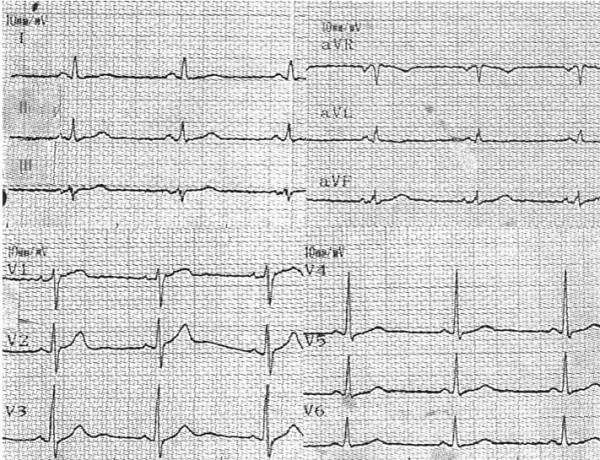
**Electrocardiogram one year later**.

A few days after her hospital discharge, the previous taken skin-muscle-vessel biopsy was proved to be diagnostic of polymyositis. Twelve months later, while being on corticosteroids, the patient remains asymptomatic, with a normal ECG (figure 4), and a normal echocardiogram.

## Discussion

Myocarditis is defined as an inflammation of the heart muscle. [1,7] Although the aetiology of myocarditis in any given patient often remains unknown, a large variety of infectious agents such as viruses, bacteria, fungi, protozoa, and even worms, a variety of autoimmune diseases, drugs, and toxins have been implicated as causative agents.[1,7-11]

Viruses are an important cause of myocarditis in North America and Europe. Infection with Coxsackie B virus specifically has traditionally percieved as the most frequent cause of viral myocarditis, causing more than half of the cases. [12] To establish the diagnosis of myocarditis due to Coxsackie B virus a distinct increase (usually fourfold) in virus-neutralizing antibodies has to be demonstrated. [8-11] In our patient, antibodies titer was not increased enough (it was detected in the upper normal limit). Consequently, the Coxsackie B4 virus could not be blamed for the disease process and the myocarditis should not be considered of a viral aetiology.

Another cause of myocarditis is the systemic collagen diseases. [7-11] Up to 40% of patients with polymyositis may have cardiac abnormalities, including AV conduction defects, tachyarrythmias, pericarditis with effusion, and a dilated, poorly contracting left ventricle. Rarely, coronary arteritis has been reported. Accordingly, the evaluation of a patient with known polymyositis who presents with chest pain, or even classic angina, and elevated cardiac enzymes, pose a diagnostic challenge. Besides an acute coronary syndrome, the differential diagnosis includes inflammatory myocarditis and coronary arteritis. [7-11] Our patient was found to suffer from polymyositis with negative examinations for viral myocarditis or coronary arteritis, as the coronary angiogram revealed normal coronary arteries. Consequently, in our case, it is quite reasonable to consider the inflammatory process due to polymyositis as the likeliest mechanism of myocarditis.

The clinical spectrum of myocarditis is very wide, ranging from asymptomatic patients to patients with severe left ventricular dysfunction or even heart failure and cardiogenic shock, as in cases of and fulminant myocarditis. Recent studies have reported only few cases of acute myocarditis mimicking acute myocardial infarction. [4-6] Differential diagnosis often requires cardiac catheterization.

Concerning the treatment, supportive care is the first line of therapy for patients with myocarditis. In patients with symptoms of heart failure, therapy should include diuretics, an angiotensin-converting – enzyme inhibitor, and a beta-blocker once clinical stability has been achieved. In some patients the intravenous administration of potent vasodilators, including nitroglycerin or sodium nitroprusside, may be required. Digoxin should be used with caution and only at low doses. In patients with severe symptoms, supportive care may include the use of intravenous inotropic therapy or implantation of a ventricular assist device. The presence of either atrial or ventricular arrhythmias may require appropriate pharmacologic therapy or possibly the implantation of a defibrillator. Bed rest should strongly be considered during the acute phase, while patients must be advised to abstain from vigorous exercise for the following months.

Concerning the usefulness of anticoagulation, most physicians nowadays suggest that, despite occasional postmortem evidence of intracardiac thrombi, anticoagulation should probably be avoided because of the risk of a hemorrhagic pericardial effusion. They recommend it only in patients with apical aneurysm with thrombus (eg, Chagas disease), atrial fibrillation, prior embolic events. [7-11] In our patient it was the presence of the mural thrombus and the direct risk of thromboembolism that forced us to the use of anticoagulants, fortunately with favorable results, rapid resolution of the thrombus without any embolic event. Our patient remained on anticoagulants for the next six months.

Two points of controversy about the treatment of myocarditis were until recently immunosuppression therapy and endomyocardial biopsy. Many clinicians believe that immunosuppression may be beneficial in patients with myocarditis and this hypothesis has been supported by a large number of uncontrolled clinical studies. However, the results of more recent randomized, placebo-controlled trials have failed to demonstrate any beneficial effects of immunosuppression. [13,14] Taken together, these studies suggest that immunosuppression should not be used in the routine treatment of patients with myocarditis. [1,8,9] Such therapy may have an important role in the treatment of patients with new onset, rapidly deteriorating, advanced heart failure with suspicion of the following conditions: giant cell myocarditis, eosinophilic, sarcoid or systemic autoimmune myocarditis. [1,7-9] Regarding myocarditis induced by polymyositis, there are no controlled trials to guide treatment decisions. In severe cases, a pulse regimen of several grams of methylprednisolone is often prescribed. Many clinicians frequently use a second agent (methotrexate, azathioprine, cyclosporine, tacrolimus) along with corticosteroids from the outset or if the patient demonstrates a chronic requirement for high dose corticosteroid therapy. Long term immunosuppressive therapy is frequently required. [11] Novel specific immunosuppressive agents, including interferon-beta, aim at interrupting myocyte injury and apoptosis and seem quite promising according to small clinical studies performed on selected patients. [10] Thus, refractory cases may respond to the addition of high intravenous immunoglobulin therapy.

Routine endomyocardial biopsy for the confirmation of myocarditis is considered unnecessary in modern literature. [1,8-10] There is a low incidence (10%) of biopsy-proven myocarditis in new onset, unexplained heart failure and false positive rates are high (50% even in four of five biopsies). Furthermore, multiple biopsies (about seven) are needed to increase sensitivity to 90%. However, endomyocardial biopsy may be considered in patients with the following conditions: a) rapidly progressive heart failure symptoms despite conventional therapy or malignant arrhythmias and b) suspected specific causes of myocarditis (eg, giant cell myocarditis).[9]

In our patient, treatment with corticosteroids was really accompanied with an apparent favorable outcome. We did not perform endomyocardial biopsy because there was a quick and progressive remission of symptoms and improvement of myocardial performance that still lasts, one year after the first appearance.

## Conclusion

Consequently, we could say that focal myocarditis mimicking acute myocardial infarction, due to polymyositis, is an unusual clinical setting to our knowledge. Clinicians should have in mind the rare possibility of myocarditis, when a patient with an autoimmune background presents with a clinical manifestation mimicking an acute coronary syndrome. The differential diagnosis may be difficult and require coronary angiography, but correct diagnosis is life – saving for the patient.

## Consent

Written informed consent was obtained from the patient for publication of this case report and accompanying images. A copy of the written consent is available for review by the Editor-in-Chief of this journal.

## Competing interests

The authors declare that they have no competing interests.

## Authors' contributions

PNS and DNC conceived the case report. PNS, DZM and AAK were involved in the case management, and drafted the manuscript. ATK and AEA performed the coronary angiography and reviewed the draft manuscript and suggested revisions. IEK and DNC reviewed the manuscript and made the final corrections before submission. All authors have read and approved the final manuscript.
